# A Journey to the Central Nervous System: Routes of Flaviviral Neuroinvasion in Human Disease

**DOI:** 10.3390/v14102096

**Published:** 2022-09-21

**Authors:** Eleanor M. Marshall, Marion P. G. Koopmans, Barry Rockx

**Affiliations:** Department of Viroscience, Erasmus University Medical Center, 3015 GD Rotterdam, The Netherlands

**Keywords:** neuroinvasion, flavivirus, blood–brain barrier, brain microvascular endothelial cells, blood–cerebrospinal fluid barrier, olfactory tract, spinal cord

## Abstract

Many arboviruses, including viruses of the *Flavivirus* genera, are known to cause severe neurological disease in humans, often with long-lasting, debilitating sequalae in surviving patients. These emerging pathogens impact millions of people worldwide, yet still relatively little is known about the exact mechanisms by which they gain access to the human central nervous system. This review focusses on potential haematogenous and transneural routes of neuroinvasion employed by flaviviruses and identifies numerous gaps in knowledge, especially regarding lesser-studied interfaces of possible invasion such as the blood–cerebrospinal fluid barrier, and novel routes such as the gut–brain axis. The complex balance of pro-inflammatory and antiviral immune responses to viral neuroinvasion and pathology is also discussed, especially in the context of the hypothesised Trojan horse mechanism of neuroinvasion. A greater understanding of the routes and mechanisms of arboviral neuroinvasion, and how they differ between viruses, will aid in predictive assessments of the neuroinvasive potential of new and emerging arboviruses, and may provide opportunity for attenuation, development of novel intervention strategies and rational vaccine design for highly neurovirulent arboviruses.

## 1. Introduction

In recent years, geographical expansion of arthropod vector populations due to climatic changes, and intrusion of human populations into sylvatic cycles of transmission as a result of increased urbanisation and population growth, has fuelled an increased risk to human health posed by arthropod-borne viruses (arboviruses) [1]. Arboviruses depend upon invertebrate vectors such as mosquitoes, ticks and sandflies for transmission between enzootic hosts, with humans often acting as incidental dead-end hosts. Many arboviruses are capable of causing severe neurological disease in humans, including members of the *Flavivirus* genus (Table 1). However, the mechanisms by which many new and emerging arboviruses gain entry to the central nervous system (CNS) to cause neurological disease, are poorly understood.

Identifying the threat posed to humans by emerging arboviruses with neuroinvasive potential is difficult because the majority of cases are mild or asymptomatic, leading to many individuals not seeking clinical care or being diagnosed with infection of a specific pathogen. In the event of severe disease or case lethality, the disease stage is too advanced to recognise initial routes of CNS invasion. In addition, regions of interest for studying neuroinvasive routes such as the choroid plexus, sciatic nerve and the olfactory epithelia, are often not investigated post mortem. Therefore, in vitro and in vivo model systems must be used to study the initial stages of disease progression and neuroinvasion and be extrapolated to the much more complex physiological setting.

The current lack of basic understanding of how, and why, arboviruses gain entry into the CNS prevents rapid identification of novel viruses with neuroinvasive potential and hinders clinical diagnoses. Therefore, the aim of this review is to provide an overview of arboviral neuroinvasive mechanisms, with a particular focus on flaviviruses, to aid the direction of future work by highlighting gaps in current knowledge and ultimately support development of targeted interventions, vaccine design and public health preparedness for current and future emerging neuroinvasive viruses.

Here, we review the two main proposed routes of arboviral neuroinvasion: the **haematogenous** and **transneural** routes.

## 2. Haematogenous Neuroinvasion

**Figure 1:**
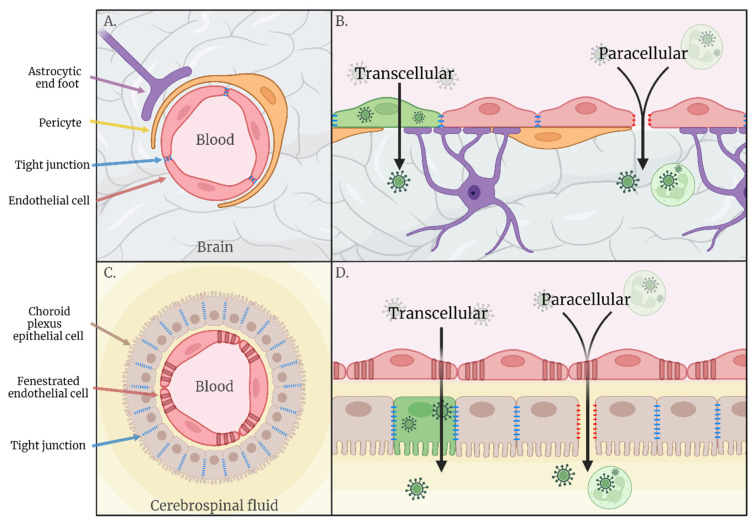
(**A**) Schematic representation of the blood–brain barrier. (**B**) Hypothesised routes of transcellular and paracellular invasion across the blood–brain barrier. (**C**) Schematic representation of the blood–cerebrospinal fluid barrier. (**D**) Hypothesised routes of transcellular and paracellular invasion across the blood–cerebrospinal fluid barrier.

Following replication at peripheral sites, such as the skin and draining lymph nodes, many arboviruses enter the blood, resulting in acute viraemia. This allows for systemic spread of infection, bringing the virus into close contact with organs distant from the initial vector bite site, including the CNS. The CNS is an immune-privileged site protected from blood-borne pathogens by physical barriers such as the blood–brain barrier (BBB). The BBB is a selective semipermeable border consisting of brain microvascular endothelial cells (BMECs), joined by a continuous line of tight junctions (TJs) and adherens junctions [2], ensheathed by astrocytes and pericytes. BMECs exhibit minimal vesicular transcytosis, limiting passage by a transcellular route [3], whilst the tight cell–cell interactions at the inter-endothelial cleft acts to limit paracellular transport. The endothelial luminal glycocalyx layer (EGL), a villiform layer of proteoglycans and glycosaminoglycans, also plays a role in vascular permeability by acting as both a physical and electrostatic charge barrier [4]. The BBB is implicated as an important interface for neuroinvasion via the haematogenous route, but research into other potential interfaces of haematogenous invasion, such as via the cerebrospinal fluid across the choroid plexus, is lacking. The endothelium of the choroid plexus does not exhibit a strict barrier function, instead the epithelial cells form tight junctions to inhibit paracellular diffusion of water-soluble molecules into the cerebrospinal fluid (CSF), establishing a blood-CSF barrier (BCSFB). Viral traverse of haematogenous barriers during viremia may occur via **transcellular** transport of virions through infected cells or via **paracellular** transport through the intercellular space between cells (Figure 1).

### 2.1. Transcellular

A prerequisite for transcellular entry into the CNS across the BBB is viral entry into BMECs. In the *Flaviviridae* family, infection of, replication within, and traversal across human BMECs has been shown in vitro for West Nile virus (WNV) [5], Japanese encephalitis virus (JEV) [6,7] and tick-borne encephalitis virus (TBEV) [8]. Only a small percentage of cells were infected [5,8], but this may be sufficient for CNS invasion and neural pathology in vivo due to the high susceptibility of neural tissue to infection [9,10]. Evidence of BMEC infection has also been identified in fatal human cases of JEV [11] and WNV [12]. However, the mechanisms of viral transport across and release from BMECs are still largely unknown.

The characteristic low rate of transcellular transport and limited vesicle formation within BMECs is, in part, due to selective expression of the MFSD2A receptor which acts to limit caveolae vesicle formation and inhibit transcytosis across endothelial cells of the CNS. The E-protein of Zika virus (ZIKV), but not WNV, has been found to specifically interact with MFSD2A leading to increased ubiquitination and degradation of this receptor both in vitro and in a neonatal mouse model [13]. However, direct evidence that a reduction in MFSD2A facilitates transcellular transport of ZIKV was not shown.

Transcytosis in absence of replication has been shown for WNV, with virus-like particles (VLPs) of the NY99 strain able to traverse human endothelial cells using a cholesterol-dependent mechanism, indicating use of lipid raft associated caveolae transport [14]. The transport of VLPs of a less virulent WNV strain, Eg101, was reduced in comparison to NY99 VLP, suggesting that endothelial cell infection and transcellular transport can be virus strain specific. A variation in the envelope protein, leading to alteration of protein structure and glycosylation, was responsible for the differing capacity for transcellular transport of the VLPs. Alterations in N-linked glycosylation of the E-protein impacts binding to the C-type lectins DC-SIGN and DC-SIGNR, which modulate the susceptibility of cells to a range of enveloped viruses [15]. The neuroinvasive capacity of Murray Valley encephalitis virus (MVEV) and JEV in mice is also attenuated by mutation of the envelope protein at distinct amino acid residues [16,17]. However, rather than facilitating transcellular invasion directly, attenuation of neuroinvasion is associated with increased glycosaminoglycan (GAG) binding. Due to the ubiquitous distribution of GAGs on cells and extracellular-matrices, enhanced binding removes virus from the blood, thereby impeding spread from extra-neural replication sites [17,18,19]. Mutations that increase virion-GAG interactions often arise in vitro as a result of cell-passage adaptation, and therefore, their role and prevalence in circulating viruses is unclear; however, naturally acquired efficient GAG binding has been suggested for arboviruses outside the *Flaviviridae* family, including Eastern equine encephalitis virus [20] and Rift Valley fever virus [21]. Characterisation of E-protein variations observed in the field may therefore still add to predictions of neuroinvasive potential for current and future emerging arboviruses [22] and targeted manipulation of GAG binding phenotypes could inform rational design of live attenuated arbovirus vaccines.

Recently, Usutu virus (USUV) has been shown to infect and traverse a human umbilical vein-derived endothelial cell model of the BBB [23] without alteration of barrier integrity [24], indicating a transcellular mode of invasion. However, an in vivo model of USUV infection has been described in which neonatal Swiss mice of less than 2 weeks of age show USUV infection of the CNS, whilst mice exceeding 2 weeks of age do not [25]. Such age-related susceptibility has also been reported for members of the alphavirus family including chikungunya virus, Semliki Forest virus, Ross-river virus and Sindbis virus [26], which may be due to the development of an intact BBB in older mice. The absence of virus in the CNS in the presence of an intact BBB suggests USUV is not transported transcellularly across the endothelial layer, seemingly contrasting results obtained from in vitro experiments [24]. However, other factors may also impact the observed age-related disparities in susceptibility in vivo, such as maturation of the immune system. Indeed, infection of adult *Ifnar*^−/−^ mice with USUV did not lead to enhanced BBB permeability but did lead to neurological disease and presence of virus in the brain [23], suggesting that the lack of neuroinvasion observed in immunocompetent models is due to the anti-viral immune control of infection, rather than an inability of the virus to invade in the presence of an intact BBB.

Transcellular passage of the BCSFB is not a well-studied route of neuroinvasion for many viruses, with the BBB often being the main focus. ZIKV was able to cross an in vitro barrier of human choroid plexus papilloma cells (HIBCPP) and human brain vascular pericytes without disruption of TJs or barrier permeability. In this model, only the pericytes, and not the HIBCPP cells that form the barrier, were susceptible to infection, indicating a transcellular mode of invasion in the absence of replication. This is supported by in vivo data in which ZIKV was found to infect choroid plexus pericytes of *Ifnar*^−/−^ mice, which led to subsequent viral presence in the CSF prior to infection of brain parenchyma [27]. Intrathecal administration of ZIKV neutralising antibodies led to a reduction in clinical signs and viral load in the brain, suggesting that the presence of cell free ZIKV in the CSF at early time points is an important contributor to the neurological disease course. Viral antigen has also been observed in the choroid plexus of mice infected with WNV [28], whilst a study of JEV tropism in a porcine model of disease showed no viral RNA or lesions in the choroid plexus [29], indicating a varying contribution of the BCSFB, and transcytosis across this barrier, to neuroinvasion by flaviviruses in these different model species.

### 2.2. Paracellular

A defining characteristic of the BBB is junctional tightness between BMECs, which limits paracellular transport of substances from the blood into the CNS [30]. Disruption of TJ proteins and increased expression of adhesion molecules leads to a decreased integrity of this barrier. During infection, the presence of key TJ proteins, such as claudin 1 and ZO1, may be reduced, despite increased or stable mRNA levels [10], suggesting perturbed localisation [31] or degradation of these proteins. Tyrosine kinases appear to have an essential role in stabilisation of TJ proteins, and therefore a complex contribution to arboviral neuroinvasion, including acting as potential entry receptors. The TAM receptor Axl has been implicated as a candidate receptor for entry of ZIKV [32] and dengue virus (DENV) [33]. An *Ifnar*^−/−^*Axl*^−/−^ model of ZIKV infection showed increased survival compared with *Ifnar*^−/−^ alone; however, viral titres in the blood and brain were similar [34], indicating entry and subsequent replication of the virus was not dependent upon Axl. Instead, the higher disease severity of animals with functioning Axl was found to stem from an increased pro-IL-1β expression and increased apoptosis of glial cells. Contrastingly, KO of TAM receptors increased vulnerability of mice to WNV, La Crosse virus (LACV) [35], and JEV [36], which was associated with impairment of BBB integrity due to defective stabilisation of endothelial TJs. These mice had a functioning type I IFN response, suggesting that the disparate results obtained in the *Ifnar*^−/−^ ZIKV model may be due to an interplay between type I IFNs and TAMs. Similarly, in addition to their influence on transcellular transport of virus across endothelial cells, Rho GTPases play an important role in the assembly, maintenance and disassembly of TJs at the inter-endothelial cleft. Hyperactivation of RhoA leads to junctional disruption, whilst Rac1 acts to down-regulate RhoA and maintain BBB function [37]. Type I IFN signalling is linked with the balanced activation of these pathways and has been shown to modulate BBB integrity in vitro by increasing localisation of TJ proteins at the cell borders of murine BMECs in response to infection [38]. BMECs isolated from wild-type (WT) mice showed rescue of BBB integrity after Th1 cytokine-mediated disruption of the barrier when subsequently infected with a low multiplicity of infection of WNV, which was not observed with BMECs of *Ifnar*^−/−^ mice. This data supports in vivo findings in which footpad (FP) inoculation of mice with defective expression of IFN-alpha (Irf7^−/−^) showed a sustained increase in BBB permeability across the entire 6 day infection course, whereas WT mice exhibited recovery of BBB integrity after 4 days [38]. However, viral titres within the brain across this time-course were not reported in this study so the effect of altered BBB integrity kinetics on neuroinvasion remains unclear. Type III IFNs play a similar role, with mice lacking the IFN-λ receptor (Ifnlr1) showing entry of WNV at earlier time-points compared to WT mice, despite similar levels of replication at peripheral sites. This rapid entry into the CNS in *Ifnlr1*^−/−^ mice was associated with an increased permeability of the BBB, corroborated by an ex vivo BBB model using BMECs of WT and *Ifnlr1*^−/−^ mice [39].

Matrix metalloproteases (MMPs) have been implicated in TJ degradation and compromise of the BBB during infection with JEV [40], TBEV [41] and WNV [42,43], and are also linked to damage of the BCSFB [44,45]. In vitro, the expression of MMPs was induced in WNV-infected human brain cortical astrocytes, most notably MMP-9, and the loss of BMEC TJ proteins could be rescued in the presence of an MMP inhibitor [42]. Further, an MMP-9*^−/−^* murine model had increased survival following WNV infection due to a decreased BBB permeability compared with WT, despite equivalent peripheral viraemia [43].

Whilst astrocytes have been implicated in release of TJ disrupting MMPs, inflammatory mediators released by microglia also play a role in the compromise of BBB permeability. JEV was found to directly interact with CLEC5A, a receptor expressed on cells of myeloid lineage [46]. In *Stat*^−/−^ mice, which are sensitive to JEV infection, blockage of CLEC5a preserved BBB integrity, reduced viral titres in the brain and inhibited immunopathology and immune cell infiltration into the CNS, leading to decreased lethality. Ex vivo microglia and mixed glial cell cultures showed that blockade of CLEC-5a did not inhibit JEV entry into or replication within these cells, but did reduce expression of the inflammatory mediators TNF-α, IL-6 and MCP-1 and attenuated neuronal damage induced by the supernatants of mixed glial cultures. CLEC5A blockade also inhibited WNV-induced activation of monocyte-derived macrophages, shown by dose-dependent inhibition of cytokine release [46]. Activation of microglia may occur as a response to local infection of the CNS; however, microglia have also been shown to contribute to the compromise of BBB integrity in response to systemic inflammation [47].

Effects of systemic inflammation on the BBB are often studied using animal models of peripheral inoculation with lipopolysaccharides [48] which have shown perturbation of the BBB due to a direct effect on the endothelial cells [49] and by activation of the brain-resident immune cells [47]. As arboviral CNS invasion occurs following initial viral replication and infection within the periphery, inflammatory effects in the brain could be induced by cytokines released into the blood from a peripheral site. Many clinical studies focus on attempting to correlate levels of proinflammatory cytokines and chemokines in the serum and CSF with the outcome of disease, in order to identify protective or detrimental mechanisms to manipulate therapeutically [50,51,52,53]. In general, elevated levels of proinflammatory cytokines and chemokines in the serum and CSF is associated with poor disease outcome, but it is not known whether this is an indicator of severe disease or actually contributory to pathogenesis. Acute-phase TBE patients have shown elevated MMP-9 in the serum and CSF [54,55], whilst patients with WNV infection exhibited elevated serum/plasma levels of a number of inflammatory cytokines [56,57] including IL-1β, TNF-α and IFN-γ [57] and MMP-9 [43] all of which have been shown to compromise the BBB [38,43,58,59,60]. Indeed, addition of sera from WNV neurological disease patients to an in vitro BBB model led to a slight increase in barrier permeability [56]. The high levels of neuroinflammatory biomarkers in these sera and the neurological clinical presentations of these patients indicates an advanced stage of neuroinvasive disease. The contribution of systemic inflammatory cytokines to the initial invasion of the CNS is therefore still unclear; however, this study does suggest that they may play a role in the progression and exacerbation of neuroinvasion via haematogenous routes. However, data obtained from in vivo and in vitro models may not always align with what is observed in the clinic. Whilst IL-1β was detected in the plasma of severe WNV disease patients, in a mouse model of WNV infection, IL-1β could not be detected in the serum at any time point tested, but could be found in the brain, indicating a local expression. This study concluded that IL-1β signalling functions to limit viral replication and load in the CNS [57], whilst others have implicated it in aiding viral neuroinvasion [59]. The role of systemic cytokines in the progression, or inhibition, of arboviral neuroinvasion and neurovirulence during human disease therefore remains unclear, and more relevant models to study this contribution must be developed.

Many host factors are implicated in modulating the integrity and permeability of the BBB in response to *Flavivirus* infection (summarised in Table 2), but specific viral factors also have a role to play. The *Flavivirus* non-structural protein NS1 has been shown to alter endothelial permeability both directly, via disruption of the EGL as a result of increased expression and activation of cathepsin L, sialidases and endoglycosidase heparinase [61,62], as well indirectly by activating immune cells, inducing release of vasoactive cytokines [63]. In vitro, this effect was found to vary between viruses and tissues, with NS1 from ZIKV, JEV, WNV, DENV or yellow fever virus (YFV) inducing different patterns of hyperpermeability in organ-specific human endothelial cells [61], which mirrors the distinct disease pathogeneses of the different flaviviruses. NS1 of WNV and JEV only bound to and induced hyperpermeability in BMECs, whilst ZIKV NS1 showed the highest binding and barrier disruption in BMECs and umbilical vein endothelial cells, suggesting that these viruses could use a paracellular mode of neuroinvasion following disruption of the BBB by NS1. These results were corroborated in vivo with administration of NS1 from ZIKV or WNV leading to increased vascular permeability in the brain of a murine model. NS1 of DENV also led to hyperpermeability of BMECs both in vitro and in vivo, to a similar extent as JEV, WNV and ZIKV. However, DENV NS1 reduced the barrier function of all organ-specific endothelial cell types tested, reflecting the systemic effects of DENV pathogenesis and showing that this effect was not BMEC specific. DENV is classified as a systemic or haemorrhagic, rather than encephalitic, *Flavivirus*, but is occasionally associated with neurological manifestations [64]. NS1 is well conserved within the *Flaviviridae* family, but does show virus-specific variation in electrostatic potential that could alter binding properties to host factors [65]. The data indeed suggests that there are virus-specific interactions of NS1 with tissue-specific surface molecules expressed by endothelial cells. Interestingly, whilst the NS1 of YFV bound to endothelial cells derived from all of the investigated organs, it only induced hyperpermeability in vitro in endothelial cells derived from human lung and liver, which was supported by increased vascular leakage in the lung and liver of the in vivo mouse model [61]. Binding of NS1 to endothelial cells alone is therefore not sufficient to induce barrier disruption, and additional mechanisms, such as induction of internalisation, may explain the tissue-specific nature of NS1 induced effects on endothelial cells. Still, the cognate attachment factor(s), the exact interactions with NS1 and the downstream mechanisms leading to increased expression or activation of EGL disrupting enzymes must still be elucidated. Further, whilst circulating NS1 has been identified in the serum of acute DENV patients [66,67], this is yet to be investigated in patients infected with the more typically neuroinvasive flaviviruses, therefore the contribution of NS1 in human disease is unclear.

Arboviruses appear to employ a number of mechanisms to disrupt the BBB, thereby opening a door for invasion into the brain. Two pathways across a permeabilised BBB have been postulated: **passive diffusion** of virions and the **Trojan horse** mechanism. This mechanism involves circumvention of the BBB by infection of, or loading onto, infiltrating leukocytes attracted to the CNS by chemokines and adhesion molecules released by activated cells of the BBB and CNS. In vitro endothelial barrier models have shown enhanced expression of certain chemokines and adhesion molecules following infection. JEV infection induced robust CINC-1, RANTES and ICAM-1 release from BMECs [6], whilst WNV also upregulates ICAM-1, along with VCAM-1 and E-selectin [5,68]. In a murine model of WNV infection, the early release of MCP-5, CXCL10 and CXCL9 indicated their role as triggers of leukocyte recruitment and infiltration [69], and blockade of CLEC5a during JEV infection reduced expression of MCP-1 and led to a reduction in infiltration of the CNS by myeloid cells [46]. Further, during MVEV infection, upregulation of the neutrophil-attracting chemokine, N51/KC, preceded infiltration of neutrophils into the CNS of MVEV-infected mice [70]. ICAM-1*^−/−^* mice exhibit a greater resistance to lethal WNV encephalitis, and a lower viral load, reduced leukocyte infiltration and decreased neuronal damage compared to controls, associated with reduced permeability of the BBB [71]. Conversely, in a diabetic mouse model with attenuated ICAM-1 and E-selectin expression, susceptibility to WNV was increased, resulting from a failure to clear WNV infection from the brain due to a reduced infiltration by leukocytes [72]. Therefore, adhesion molecules appear to play a contrasting role in facilitation of viral neuroinvasion and recruitment of immune cells to clear the virus from the brain.

The expression of adhesion molecules, chemokines and inflammatory factors that impact the permeability of the BBB leads to an environment in which the BBB is permissible to leukocytic infiltration [68,71], making viral infection of leukocytes a potential mechanism to increase viral load within the CNS via the Trojan horse mechanism. In vitro data indicates that many immune cells are susceptible to infection with arboviruses, but this may not correlate with the ability of these cells to be infected in the periphery and subsequently traffic into the CNS to establish, or contribute to, infection in vivo. For example, in the case of JEV, monocytes are susceptible to infection in vitro, but no viral antigen could be identified in perivascular cell infiltrates of JE patients [11] and PBMCs of an in vivo porcine model did not show infection with JEV [29]. So, what evidence exists of the ability of arboviruses to traffic within or bound to immune cells? Evidence of WNV-infected leukocytes within the CNS has been shown [71], but there is a lack of data to indicate that peripherally infected leukocytes traffic to and traverse into the CNS. Splenic T cells are permissive to WNV infection, and brain-infiltrating T cells show staining for WNV antigen [73]. However, in this study, brain infiltrating T cells were isolated at a late time point of the infection course, so the infiltrating cells could have been infected in the brain rather than the periphery.

Osteopontin (OPN) is a protein expressed by many immune cells that contributes to recruitment of polymorphonuclear cells (PMNs) into the brain and stabilisation of the BBB via MAPK-mediated pathways [74]. However, KO of OPN in WNV-infected mice led to a less permeable BBB, reduced viral load and PMN infiltration in compared to WT [75]. Disruption of the BBB seen in the WT mice could be due to neuroinflammation secondary to an established infection within the brain as a result of an increased early influx of (infected) immune cells compared with OPN*^−/−^*. However, the presence of infected PMNs within the brain is not necessarily proof that these cells were infected prior to infiltration, but instead they may have been infected after entry into the CNS, and therefore, the initial route of invasion is still not clear. In any case, OPN appears to contribute to the paradoxical role played by the immune system during neuroinvasive disease, and indicates that paracellular mechanisms of neuroinvasion may contribute to WNV neuropathogenesis. A PMN predominance within infiltrating cell populations has also been shown during MVEV infection of a neonatal mouse model, in which depletion of neutrophils led to a prolonged survival and reduced mortality of infected mice compared to infected controls; however, similar viral titres were observed in the brain [70]. It is therefore likely that the attenuation of disease does not stem from reduced MVEV invasion of the CNS within infected neutrophils, but it is due to a reduction in inflammatory-mediated pathology.

## 3. Transneural Neuroinvasion

**Figure 2:**
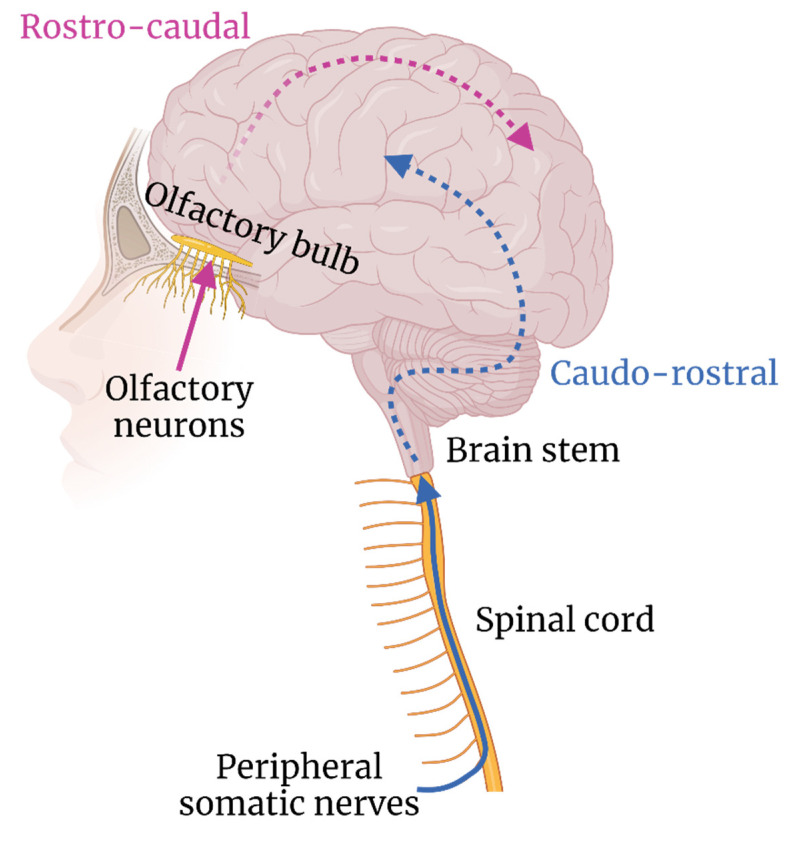
Hypothesised routes of transneural neuroinvasion and subsequent neuron–neuron spread.

Infiltration of the CNS along transneural pathways is known for a number of viruses, including rabies virus, poliovirus and herpes simplex virus (HSV). However, research into arboviral infiltration of the CNS via transneural routes is comparatively limited. Two neuroanatomical areas have been postulated to be involved in CNS invasion, namely: **peripheral nerves** and **olfactory nerves** (Figure 2).

### 3.1. Peripheral Nerves

Skeletal muscles are innervated by peripheral motor nerves projecting from the anterior grey column of the spinal cord, which acts as a direct ascending pathway into the brain via the brain stem (Figure 2). Arboviruses have been implicated in using this pathway, with injury to the anterior horn motor neurons of the spinal cord observed in patients with neuroinvasive WNV infection, leading to acute flaccid paralysis with associated muscle weakness [78,79]. Direct injection of WNV into the sciatic nerve of a hamster model led to limb paralysis, which was blocked by axotomy of the sciatic nerve. However, an axotomy did not prevent spread of WNV into the CNS via an assumed alternate route [80]. Using a similar model, WNV was found to show a preference for transport along motor axons of the sciatic nerve, rather than sensory axons, leading to damage of the spinal cord, motor weakness and paralysis [81]. The underlying determinants of tropism for motor but not sensory neurons, remains to be determined. Evidence of anterior horn cell involvement has been shown in patients with JEV, TBEV and MVEV infection [82,83,84]. TBEV especially appears to show a preference for infection of the anterior horn cells of the cervical spinal cord [85,86]; however, the route by which these cells come to be infected is not known. Infection resulting from transport along peripheral nerves is a possibility, but the plentiful blood supplied to these cells via the sulcal branches of the anterior spinal artery also provides a haematogenous route of infection.

Viral spread via neurons can occur via bi-directional axonal transport. In vitro data, using compartmentalised neuron cultures, revealed a bidirectional transport of WNV along and between neurons. Intact axons were required for intraneuronal spread of WNV, indicating a transmission of virus across synapses [80]. A light chain of human dynein, associated in viral transport of HSV [87] and polio virus [88], was found to interact with the M protein of WNV, JEV and DENV [89]. The hypothesis that WNV can be transported by membranous microtubule-mediated transport is strengthened by the finding that treatment with a microtubule inhibitor during in vivo WNV infection of the sciatic nerve significantly reduced WNV staining in the lumbosacral spinal cord, indicating attenuated axonal spread [81].

### 3.2. Olfactory Nerves

The olfactory pathway consists of unmyelinated olfactory neurons that branch from neuroepithelium lining the nasal cavity, and enter the CNS via the cribriform plate to synapse with cells of the olfactory lobes [90], thereby acting as a direct route into the brain. Detection of WNV, JEV and MVEV in the olfactory bulb of in vivo infection models at early timepoints has led to the hypothesis that flaviviruses can gain access to the CNS using the olfactory nerves [11,29,91,92,93]. Trans-olfactory neuroinvasion may suggest a route of transmission other than via an arthropod bite. In a porcine model of JEV infection, known to act as amplifying hosts for this virus [94,95], neuroinvasive disease occurred following direct contact with infected pigs, as well as following oronasal inoculation [96]. Intranasal inoculation led to detection of JEV antigen in the olfactory bulb, with glial cell aggregation and perivascular cuffing throughout the olfactory tract [97]. In another study by the same authors, fewer lesions and reduced viral titres in the olfactory bulb were observed compared to other brain areas at days 7 and 11 post infection, which was consistent across viral dosage and route of inoculation [96]. The wide range of brain regions involved implicates the haematogenous route of invasion for JEV entry into the CNS, rather than trans-olfactory. As these time points represent an advanced stage of disease, sampling at earlier points would help to elucidate the contribution of transient olfactory bulb infection in this model. Indeed, a further study, using intravenous inoculation, found that at only 3 days post infection, the nasal epithelium and olfactory neuroepithelium had the highest viral titres [98], indicating that the contribution of the olfactory route to neuroinvasion may be transient and occur early in the disease course.

The spread of MVEV strains of high (BH3479) and low (BHv1) neuroinvasive potential in a Swiss mouse model [99] after peripheral inoculation, showed entry into the CNS via the olfactory nerves. Both strains were identified within the olfactory lobes prior to infection of other brain regions; however, the low pathogenicity strain was restricted to this area, showed reduced titres compared with BH3479 and had significantly lower levels and persistence of viraemia. A rostro-caudal dispersion of BH3479 has been observed [99], suggesting direct spread of virus from the olfactory bulb to wider brain regions. Despite convincing experimental data, MVEV presence within the olfactory tract and olfactory bulb has not been detailed in clinical cases [100,101,102]; however, this may be a result of a lack of sampling or the advanced stage of disease sampled.

A mouse model of WNV progression showed that at only 3 days post infection, there were high viral titres in both the spinal cord and olfactory bulb compared with other brain areas [92]. As there was no significant difference in viral loads between the spinal cord and olfactory bulb, it is possible that transneural neuroinvasion can occur concurrently at spatially distant sites. However, in fatal cases of WNV virus, lesions and viral antigen are most commonly observed in the brainstem and anterior horns of the spinal cord, suggesting invasion from ascending peripheral nerves or a haematogenous route, rather than olfactory.

### 3.3. Other Possible Routes of Transneural Invasion

TBEV can be transmitted via the alimentary route by drinking raw milk products from infected livestock [103,104], and retains infectivity following exposure to the low pH environment of the stomach. Intestinal epithelial cells are first infected before viral entry to the intestinal lymphoid tissue [105], but the mode of subsequent progression towards the CNS is poorly understood. Recently, evidence of a gut–brain neural circuit has emerged in which enteroendocrine cells of the mouse gut form synapses with vagal neurons, providing a direct signalling pathway from the gut to the brain [106]. WNV has also been found to replicate in the intestines of a mouse model [92] and has tropism for enteric neurons [107], which may contribute to the symptoms of gastrointestinal distress and dysfunction observed in human infections [92,108]. The contribution of alimentary infection and transneural invasion via the gut–brain neural circuit to arboviral neuroinvasion is yet to be elucidated

The eye is an immune privileged site that exhibits barriers with the blood, broadly titled the blood–ocular barrier (BOB), similar to that of the BBB. Neuroinvasion via the retinal ganglions has been suggested for some viruses [109,110,111], but little research has been conducted regarding the use of this pathway by arboviruses. Clinically, severe ophthalmic impairment has been reported for many neuroinvasive arboviruses including WNV [112] and JEV [113], often presenting with neuritis, immune cell infiltration or abnormality of the BOB, potentially allowing dissemination of virus from the blood into the eye. In an experimental setting, peripheral inoculation of an *Ifnar*^−/−^ mouse model with USUV led to severe ocular defects including neuroretinitis and uveitis, and infiltration of microglia, with similarly high viral titres observed in the eye and brain [23]. However, ZIKV and DENV are also associated with a range of clinical ocular disease states [114], but they rarely induce neurological disease in immunocompetent adults, indicating that despite ophthalmic involvement, neuroinvasion via the optic nerve likely does not occur for these viruses.

## 4. Conclusions and Future Perspectives

The increased number, frequency and geographical distribution of neurotropic arbovirus outbreaks in recent years has led to an urgent need for a greater understanding into the tendency of many arboviruses to invade the CNS and how this process can be modulated. The BBB has been a central focus of research for many neuroinvasive viruses, but direct evidence of invasion across this barrier via the Trojan horse mechanism is not well established, and more research into the trafficking behaviour of arbovirus-infected immune cells is required. In addition, Trojan horse invasion across the BCSFB is a largely unfilled gap in our current understanding of arboviral neuroinvasion, but is especially relevant when considering that CSF pleocytosis is a common diagnostic indicator of viral meningoencephalitis. Indeed, compared with the BBB, the other haematogenous barriers, including the BCSFB and the BOB, have so far been neglected in the field of arbovirology. Multidisciplinary development and application of human relevant in vitro model systems, and an increased focus on these barriers in vivo and at autopsy, would aid in closing this gap. For example, an organoid model of the BCSFB, developed in the field of neurodevelopmental biology, was recently applied to study SARS-CoV-2 tropism and pathogenesis [115]. This model system could also be applied to identify the contribution of the BCSFB to arboviral neuroinvasion.

The primary route of arboviral transmission is via the bite of an arthropod vector. However, alternate routes of transmission do exist. Further investigation into how the route of transmission may influence the route of neuroinvasion could allow for identification of novel preventative and therapeutic strategies. The gut–brain neural circuit has not yet been studied as a route of transneural arboviral invasion but could be relevant for arboviruses with gastro-intestinal involvement, especially if a virus, such as TBEV, can be transmitted via the alimentary route. Similarly, the proposed oronasal transmission of JEV indicates a direct route of CNS invasion along the olfactory tract. However, experimental work studying the trans-olfactory route of invasion must aim to identify a progression of infection along the olfactory tract over time, rather than relying on the presence of virus in the olfactory bulb alone.

**Figure 3:**
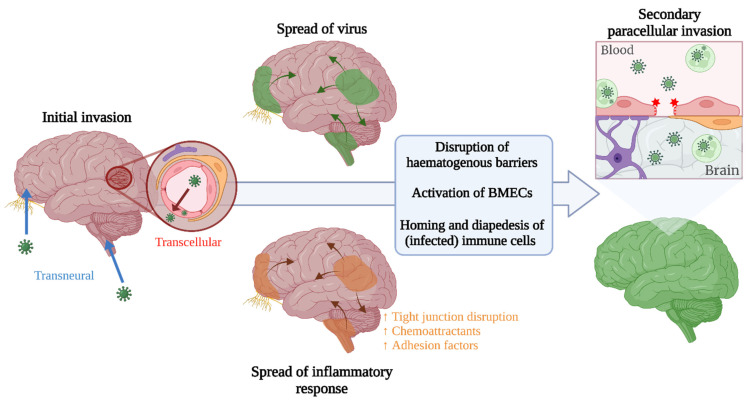
Speculative representation of how neuroinvasive routes could be interlinked across the course of disease, leading to increased viral presence in the brain following an initial invasion via an alternate route.

The viruses discussed show variation in their capacity for invasion of the CNS and the mechanism by which this is achieved (summarised in Table 3), and are rarely studied side by side. Yet, an overarching commonality between them is the interconnectedness of the routes of neuroinvasion. Many arboviruses have been implicated in using both haematogenous and transneural routes of neuroinvasion, but the spatio-temporal kinetics of these multi-pronged mechanisms, and the interdependency between the different routes of invasion, are largely unknown (Figure 3). Further delineation of these factors, using in vivo serial sacrifice studies combined with route-specific manipulation of invasion, would aid in identification of more effective intervention strategies, as therapeutic modulation of only one route of neuroinvasion may not be sufficient to prevent neurological disease resulting from invasion by another route. Additionally, the antiviral immune response has a complex influence on the progression and severity of neuroinvasive disease, on one hand facilitating control and clearance of infection, and on the other, potentially contributing to disease severity due to immune-mediated pathology, BBB disruption, and the Trojan horse mechanism of neuroinvasion. Infiltration of immune cells into the CNS usually only occurs following development of an proinflammatory environment therein, suggesting that prior infection of the CNS is required, but migration of highly activated immune cells across a non-inflamed BBB has been shown in experimental in vivo studies of autoimmune encephalitis [116]. The contribution of the Trojan horse mechanism to initial seeding of infection within the CNS across steady-state haematogenous barriers is therefore another gap to be filled. Furthermore, systemic inflammation could also have a role in facilitating neuroinvasion and, in the context of arboviral infection, many specific immune mediators have been identified in the serum and CSF of patients that are correlated with severe neurological disease. Deeper understanding into the role of arbovirus-specific local and systemic immune responses, and the balance between protection and pathology, could support development of safe and effective immune-directed interventions. As an additional complication, in many geographical regions, numerous (arbo)viruses co-circulate. The contribution of co-infection and pre-existing immunity to the method and progression of arboviral neuroinvasion and pathogenesis, is yet another important dimension to unravel in both experimental and clinical settings. Further investigation is also required into the factors that influence host susceptibility to neuroinvasive disease. Advanced age and comorbidities, such as hypertension and diabetes, have already been implicated in increasing risk of neurological disease induced by arboviruses [117], but the underlying mechanisms and the genetic or lifestyle factors contributing to this have not been elucidated. Epigenetic mechanisms may also play a role, with tissue-specific epigenetic modifications shown to influence the relative expression of interferon stimulated genes in certain brain regions and thereby affect the susceptibility of neuronal subtypes to infection [118], but how epigenetics may fit into the wider picture of host susceptibility is a gap to be filled.

Extrapolating what is known about well-studied arboviruses, such as WNV, to predict the neuroinvasive capacity of closely related emerging viruses is an attractive concept. However, as demonstrated by WNV and USUV, even closely related viruses can display a disparate capacity to cause neurological disease in humans. Understanding the root of mechanistic differences will therefore further aid in predictive assessments of neuroinvasive potential, provide opportunity for attenuation and discovery of therapeutic targets, and allow development of platforms for rational vaccine design and vaccine safety assessment for highly neurovirulent arboviruses.

## Figures and Tables

**Table 1:** Flaviviruses known to cause neurological disease in humans.

Family	Genus	Species
*Flaviviridae*	*Flavivirus*	*West Nile virus*
		*Usutu virus*
		*Japanese encephalitis virus*
		*Saint Louis encephalitis virus*
		*Murray Valley encephalitis virus*
		*Ilheus virus*
		*Zika virus*
		*Wesselsbron virus*
		*Dengue virus*
		*Powassan virus*
		*Tick-borne encephalitis virus*

**Table 2:** Summary of the host factors discussed in this review and their contribution to the initial invasion of the CNS by flaviviruses.

			Role	
Type	Factor	Virus	Increase Invasion	Decrease Invasion
Adhesion molecules	E-selectin	WNV [5]	Increases recruitment of leukocytes to the BBB and CNS and enhances attachment of leukocytes. Allows for potential Trojan horse and further release of inflammatory cytokines that impact haematogenous barrier integrity.	
	ICAM-1	JEV [6] WNV [5,71]		
	VCAM-1	WNV [5]		
Chemokine	CINC-1	JEV [6]		
	CXCL9	WNV [69]		
	CXCL10	WNV [69]		
	MCP-1	JEV [46]		
	MCP-5	WNV [69]		
	N51/KC	MVEV [70]		
	Osteopontin	WNV [75]		
	RANTES	JEV [6]		
Cytokine	IFN-γ	WNV [38]	Activates and disrupts the BBB.	
	IL-1α	JEV [76]		
	IL1-β	ZIKV [34]		
	TNF-α	WNV [60]		
Enzyme	Cathepsin L	ZIKV JEV WNV DENV [61]	Disrupts EGL layer of brain endothelium [61].	
	Endoglycosidase heparinase			
	Sialidases			
	MMP-9	JEV [40], TBEV [41] WNV [42,43]	Involved in degradation of BBB TJ proteins [42,43].	
	RacA			Down regulates RhoA [37].
	RhoA		Hyper activation leads to junctional disruption [37].	
Receptor	CLEC5a	JEV WNV [46]	Blockage preserved BBB integrity [46] indicating role in BBB dysregulation via induction of inflammatory mediator release [46].	
	GAGs	WNV [14]. JEV MVEV [16,17]	Attachment receptor [14,15].	Increased GAG binding sequesters virus in periphery [17,18,19].
	IFNAR	WNV [38]		Balances RhoA-RacA activation [37]. Increases localisation of TJ proteins at endothelial cell border [38].
	IFNLR1	WNV [39]		KO increases permeability of BBB indicating role in BBB maintenance [39].
	MFSD2A	ZIKV [13]	Inhibits vesicular transcytosis across BMECs [77] and is ubiquitinated by ZIKV E protein binding [13].	
	TAM	ZIKV [32] DENV [33] WNV [35] JEV [36]	Candidate entry receptor for ZIKV and DENV [32,33].	Involved in stabilisation of endothelial TJs [35,36].

**BBB** = blood–brain barrier. **CINC-1** = cytokine-induced neutrophil chemoattractant 1. **CLEC5a** = C-type lectin domain containing 5A. **CXCL10** = C-X-C motif chemokine ligand 10. **CXCL9** = C-X-C motif chemokine ligand 9. **DENV** = dengue virus. **EGL** = endothelial glycocalyx layer. **GAG** = glycosaminoglycan. **ICAM-1** = intercellular adhesion molecule 1. **IFNAR** = interferon-α/β receptor. **IFNLR1** = interferon lambda receptor 1. **IFN-γ** = Interferon gamma. IL-1α = interleukin 1 α. **IL-1β** = interleukin 1β. **JEV** = Japanese encephalitis virus. **KO** = knockout. **MCP-1** = monocyte chemoattractant protein-1. **MCP-5** = monocyte chemoattractant protein-5. **MSFD2A** = major facilitator superfamily domain containing 2A. **MMP-9** = matrix metalloproteinase-9. **MVEV** = Murray Valley encephalitis virus. **RANTES** = regulated upon activation, normal T cell expressed and secreted. **TAM** = TYRO3, AXL and MER. **TBEV** = tick-borne encephalitis virus. **TJ** = tight junction. **TNF-α** = tumour necrosis factor α. **VCAM-1** = vascular cell adhesion molecule 1. **WNV** = West Nile virus. **ZIKV** = Zika virus.

**Table 3:** Summary table of the potential modes of CNS invasion used by the neurotropic flaviviruses discussed in this review. ✓ = supported by experimental data. X = not supported by experimental data. ? = suggested or yet to be demonstrated. * = also includes differentiated iPSC and CD34+ cord blood-derived BMEC-like in vitro cell models.

	Haematogenous					Transneural			
Virus	Infection of BMECs *	Transport across BMECs *	Trojan Horse	Increased BBB Permeability	Choroid Plexus	Olfactory	Spinal Cord	Gut–Brain	Optical Nerve
JEV	✓ [6,119]	✓ [119]	X [11,29]	✓ [31,46,61,120]	X [29]	✓ [29,97,98]	? [121]	?	? [113]
TBEV	✓ [8]	✓ [8]	?	✓ [41,122,123]	?	? [124]	? [85,86]	? [103,105]	? [125]
USUV	✓ [23]	✓ [23]	?	X [23]	?	? [126]	? [23]	?	? [23]
WNV	✓ [5,12]	✓ [5,14]	✓ [71,73]	✓ [10,42,43,61]	✓ [28]	✓ [91,92,93]	✓ [78,79,81,92]	? [92,107]	? [112]
ZIKV	✓ [13]	✓ [127]	✓ [128,129]	✓ [61]	✓ [27]	? [130,131]	? [132]	?	? [114]

**BBB** = blood–brain barrier. **BMEC** = brain-microvascular endothelial cell. **JEV** = Japanese encephalitis virus. **TBEV** = tick-borne encephalitis virus. **USUV** = Usutu virus. **WNV** = West Nile virus. **ZIKV** = Zika virus.

## Data Availability

Not applicable.
